# Pak1 Regulates the Orientation of Apical Polarization and Lumen Formation by Distinct Pathways

**DOI:** 10.1371/journal.pone.0041039

**Published:** 2012-07-18

**Authors:** Orlando deLeon, Jason M. Puglise, Fengming Liu, Jos Smits, Martin B. ter Beest, Mirjam M. Zegers

**Affiliations:** 1 Department of Surgery, University of Chicago, Chicago, Illinois, United States of America; 2 Genitourinary Medical Oncology UT MD Anderson Cancer Center, Houston, Texas, United States of America; 3 Department of Cell Biology, NCMLS, Radboud University Nijmegen Medical Center, 6525 GA Nijmegen, The Netherlands; University of Birmingham, United Kingdom

## Abstract

The development of the basic architecture of branching tubules enclosing a central lumen that characterizes most epithelial organs crucially depends on the apico-basolateral polarization of epithelial cells. Signals from the extracellular matrix control the orientation of the apical surface, so that it faces the lumen interior, opposite to cell-matrix adhesion sites. This orientation of the apical surface is thought to be intrinsically linked to the formation of single lumens. We previously demonstrated in three-dimensional cyst cultures of Madin-Darby canine kidney (MDCK) cells that signaling by β1 integrins regulates the orientation of the apical surface, via a mechanism that depends on the activity of the small GTPase Rac1. Here, we investigated whether the Rac1 effector Pak1 is a downstream effector in this pathway. Expression of constitutive active Pak1 phenocopies the effect of β1 integrin inhibition in that it misorients the apical surface and induces a multilumen phenotype. The misorientation of apical surfaces depends on the interaction of active Pak1 with PIX proteins and is linked to defects in basement membrane assembly. In contrast, the multilumen phenotype was independent of PIX and the basement membrane. Therefore, Pak1 likely regulates apical polarization and lumen formation by two distinct pathways.

## Introduction

Many organs develop by organizing epithelial cells into a basic architecture of branching tubules enclosing a central lumen. A hallmark of the cells surrounding these lumens is apico-basolateral polarization. Typically, cells have an apical surface that faces the interior of the lumen. The basolateral surface comprises a lateral and a basal domain, which mediate adherence to neighboring cells and the underlying extracellular matrix (ECM), respectively, via different adhesion complexes. At the lateral surface these include tight junctions, which separate the apical and basolateral domains, whereas E-cadherin-based adherens junctions mediate cell-cell adhesion. Integrin-based focal adhesions at the basal surface mediate adhesion to the ECM.

Cell-matrix and cell-cell adhesion complexes not only mediate cell adhesion, but are also important signaling centers that are critical to generate and maintain apical and basolateral polarization [1], [2]. Cell polarization is crucial for maintaining tissue homeostasis and polarized 3D tissue organization, and may serve as a non-canonical tumor suppressor [3]. Three conserved protein complexes play a central role in the establishment and maintenance of apico-basolateral cell polarization [2]. The Crumbs-Pals1-Patj and the Par3-Par6-atypical PKC (aPKC) complexes localize apically and promote the identity of the apical domain. The Lethal giant larvae-Scribble-Discs large complex at the basolateral surface defines basolateral identity. The apical and basolateral polarity complexes appear to function in a mutually exclusive manner, and in concert regulate the size of, and boundary between, the apical and basolateral membrane domains. It was suggested that the correct orientation of the apical surface is intrinsically linked to the ability of epithelia to form single lumens [4], [5]. Indeed, the loss-of-function of either of the three polarity complexes inhibits the formation of a single lumen and generally leads to a multilumen phenotype [2].

The Madin-Darby canine kidney (MDCK) cell line has been extensively used as a model system to study epithelial polarization and lumen formation. Historically, cell polarization has mostly been studied in two-dimensional (2D) culture, such as culture on semi-permeable filter supports. A drawback of these models is that they are anisotropic, meaning that these supports provide a strong polarizing cue. This cue is often sufficient to drive the orientation of the apical surface [1], thus precluding the analysis of how the orientation of the apical domain is regulated. In three-dimensional (3D) culture, single cells suspended in a gel of purified collagen or extracellular matrix (ECM) extract, proliferate to form fluid-filled cysts consisting of a monolayer of polarized cells enclosing a lumen. The isotropic environment of 3D models has been instrumental in deciphering pathways that control orientation of polarization [6]. Signals from the ECM, and in particular the laminin-rich basement membrane (BM), are crucial to establish apical polarization [7]. Pathways involving β1 integrin-mediated activation of the Rho GTPases Rac1 and cdc42 play a central role in this process. β1-integrins activate Rac1 in MDCK [8] and many other cells [9], [10], and β1 integrins [11], [12], [13] and Rac1 [8] are required to form apical surfaces. We previously showed that inhibition of β1 integrin [8], [14] or Rac1 signaling [15] leads to the formation of cysts in which the orientation of the apical surface is inverted, in that it faces the ECM-cell interphase. The inverted orientation of polarity of these cysts is due to an inability to properly assemble laminin at the cyst periphery [8], [15]. Presently, the responsible Rac effector molecules mediating this phenotype have not been identified.

Pak1 is one of the best-characterized effector proteins of Rac and cdc42 [16]. It belongs to the highly conserved group A of Pak family kinases (Pak1, Pak2 and Pak3), which are activated upon binding of activated Rac or cdc42. Activated Rac and cdc42 also bind the related group B Paks (Pak4, Pak5 and Pak6), but this does not appear to affect the kinase activity of these Paks [17]. Pak2 and Pak4 have been implicated in lumen formation in endothelial cells via a mechanism that also relies on the polarity complex proteins Par3 and aPKC [18]. In the Drosophila salivary gland, constitutive activation of Pak1 inhibits the formation of a single apical lumen [19]. Furthermore, Pak1 may control apical-basolateral polarization of the Drosophila follicular epithelium in a signaling pathway linking the signals from the basement membrane to polarity complex proteins such as Crumbs [20].

Here, we investigated whether Pak1 controls the orientation of the apical surface and lumen formation. We find that active Pak1 misorients the apical surface and controls the formation of single lumens. Our data indicate that these two phenotypes are mediated by different pathways. Specifically, we find that orientation of polarization is directly linked to cell-ECM interactions, whereas lumen formation is mediated by a ECM-independent pathway. Together, our data show that Pak1 is an important regulator in maintaining tissue architecture.

## Methods

### Antibodies and other Reagents

Mouse monoclonal antibodies (mAb) to anti-phosphorylated FAK (pY397-FAK), polyclonal rabbit anti-β-catenin, anti-paxillin and anti-PKCζ antibodies were purchased from Santa Cruz Biotechnology, Inc. Mouse anti-βPIX and anti-β1 integrin mAbs were from BD Biosciences. Rat anti-β1-integrin function blocking antibodies were from Developmental Studies Hybridoma Bank, University of Iowa. Mouse monoclonal anti-β-tubulin and rabbit anti-laminin antibodies were from Sigma-Aldrich. Rabbit anti-MEK and pS298-MEK were from Cell Signaling. Mouse anti-gp135/podocalyxin antibodies were a gift from George Ojakian. Secondary antibodies comprised Alexa Fluor™ 488/555 donkey anti-mouse IgG (H+L) and anti-rabbit conjugates and Alexa Fluor™ 488/633 goat anti-rat IgG (H+L) conjugates (Invitrogen). Chemicals were from Fisher Scientific.

### Cell Culture and Cell Lines

Myc-tagged CA-Pak1 in pCMV6M (provided by Jonathan Chernoff) was subcloned into the vector pCDNA6V5/HisA (Invitrogen) in which the CMV promoter was replaced with the CMV-tet promoter. The CA-Pak1ΔPIX mutant was made by excising the Pak1 fragment harboring the R193G and P194A mutations in the PIX binding domain from the Pak1-K299R,R193G,P194A mutant in pCMV6M [21] and switching it with the same region of CA-Pak1-pCDNA6V5/HisA-CMV-tet. The final plasmid DNA sequence was verified by sequencing. MDCK cells were transfected with these constructs using the calcium phosphate co-precipitation method and myc-expressing clones were selected using 6 µg/ml blasticidin S hydrochloride (Invitrogen). Blasticidin-resistant clones were isolated by ring cloning.

Cells were grown in modified Eagle’s Medium (MEM) with L-glutamine, supplemented with 10% fetal calf serum and 1% penicillin/streptomycin. To inhibit gene expression, cells were maintained in growth medium with 20 ng/ml doxycycline. Doxycycline was removed by washing the cells three times with PBS followed by addition of growth medium to initiate induction of gene expression. Cells were induced for 2–3 days before plating on 12 mm 0.4-µm pore polycarbonate Transwell filters (Corning-Costar), glass coverslips (for focal adhesion staining), or as 3D culture in a collagen I matrix or reconstituted BM extract (BME) [22], [23], [24].

### siRNA

Pak siRNAs were Stealth siRNA (Invitrogen) and comprised the following targeting sequences: Pak1 KD-1:ggaugcggcuacaucuccuauuuca; Pak1 KD-2: cagccgaagaaagagcugauuauua. Scrambled Stealth oligonucleotides were used as controls for Pak1 knockdown experiments. For transient transfections 4×10^6^ cells were electroporated with 100 pmoles of siRNA, using an Amaxa nucleofector (program T23, buffer T) (Lonza).

### Immunoprecipitation

Cells grown on 10 cm dishes were washed twice with cold PBS, lysed in cold immunoprecipitation (IP) lysis buffer (125 mM NaCl, 20 mM Hepes pH 7.4, 1% Igepal) containing protease and phosphatase inhibitors (1 mM Na_3_VO_4_, 1 mM NaF) and scraped from the plate. Lysates were precleared with Sepharose CL-4B beads (GE Healthcare, Piscataway, NJ) and protein concentrations were determined by a bicinchoninic acid assay (Pierce Biotechnology). Samples were normalized to 800–1000 µg protein and primary antibodies were added to cell lysates. As negative controls, anti-mouse or anti-rabbit IgG antibodies (Pierce Biotechnology) were added. Samples were rotated for 1 hour at 4°C. Next, 40 µl of 12.5% washed Sepharose-Protein A or Sepharose-Protein G was added and samples were incubated overnight at 4°C while rotating. The beads were washed with IP lysis buffer and eluted from the beads by boiling for 5 minutes in Laemmli lysis buffer. The immunoprecipitates, along with a sample of the total lysate, were analyzed by SDS-PAA gel electrophoresis and Western blotting.

### Rho GTPase Assay

Levels of RhoA-GTP were determined using a Rhotekin-RBD pulldown assay as previously described for cell culture in 2D [25] and 3D [14].

### Western Blotting

Unless indicated otherwise, cell lysates were prepared from confluent cells grown for 6 days on filters. Cells were washed and lysed in lysis buffer containing 1% SDS. Equal amounts of protein were diluted in 2x Laemmli buffer, loaded onto SDS-PAA gels, and transferred onto PVDF membrane (Millipore). Quantitative Westerns were performed using an Odyssey detector (LI-COR, Lincoln, NE). Secondary antibodies for Odyssey detection comprised Alexa Fluor IRDye 800-conjugated or Alexa Fluor 680-conjugated donkey anti-mouse and donkey anti-rabbit IgG (Invitrogen). Secondary antibodies for Western blots stained for pS298-MEK comprised HRP-conjugated donkey anti rabbit IgG (Jackson Immunoresearch Laboratories) and blots were visualized by enhanced chemiluminescence (ECL, GE Healthcare).

**Figure 1:**
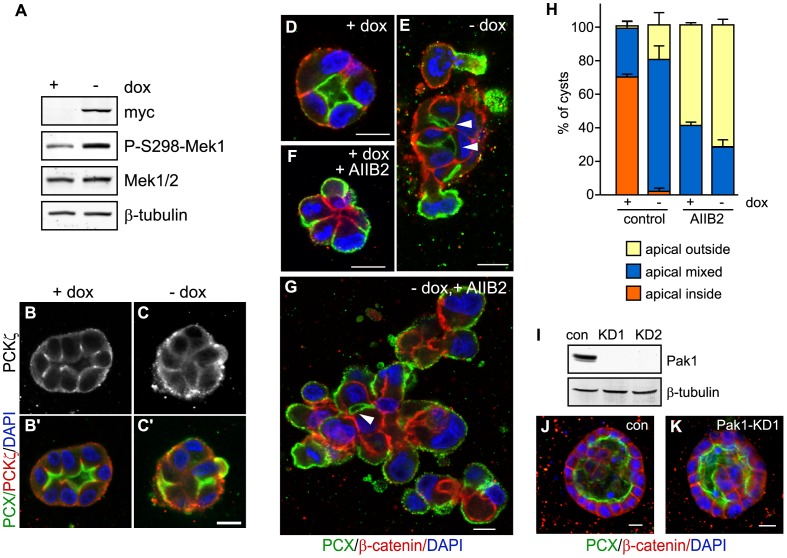
Constitutive active Pak1 misorients the apical surface and inhibits single lumen formation in 3D culture. MDCK cells inducibly expressing myc-tagged CA-Pak1 under control of the Tet-off system were grown with (control, +dox) or without dox (CA-Pak1 expression, -dox). **A**: Lysates from four day old cells plated at confluent densities on Transwell filters were analyzed by Western blot and show expression of CA-Pak1 (myc), phospho-S298-Mek1, total Mek1/2. β-tubulin is the loading control. **B–C’**: Control (B,B’) and CA-Pak1-expressing cells (C,C’) were grown for four days to cyst or spheroids in 3D collagen I culture and stained for PKCζ (white in B,C, red in B’,C’) and PCX (green in B’,C’). **D–G**: show control (D,F) or CA-Pak1-expressing cells (E,G) grown in 3D collagen in the absence (D,E) or presence (F, G) of the β1 integrin function blocking antibody AIIB2. Cells were stained for PCX (green) and β-catenin (red). Small apical lumens in CA-Pak1-expressing cells are indicated by arrowheads. **H**: Quantification of phenotypes at conditions shown in D–G. n = 3. A typical image of apical inside (orange) is shown in figure D, apical outside (yellow) is shown in F, and apical mixed (blue) in E and G. **I–K**: Parental MDCK cells were transiently transfected with a scrambled siRNA control (con) or siRNA’s targeting Pak1 (Pak1-KD1, KD2). I: Western blot showing Pak1 knockdown by two different siRNA’s. J,K show cysts stained for PCX (green) and β-catenin (red). Nuclei in B’–G, J–K are blue, scale bars are 10 µm.

### Trypsin Protection Assay

Cells were plated at confluent density on 12 mm filters and maintained in culture for 6 days. Cells were washed with PBS with 1 mM calcium and 1 mM magnesium (PBS^++^) and then incubated with 7.5 mg/ml trypsin (TRL3, Worthington) in PBS^++^ for 30 min at 37°C to digest extracellular proteins and proteins expressed at the plasma membrane. As positive control for digestion, 0.1% Triton X-100 was added to the trypsin solution to permeabilize the membrane and to digest all cellular protein. Staining of intracellular tubulin was used as a control for protection against trypsin digestion. After incubation, detached cells in the apical medium were collected by centrifugation, and were pooled with the remaining cells and matrix on the filter. The pooled samples were lysed in lysis buffer containing 1% SDS. Mock-treated controls were kept in PBS^++^ for 30 minutes at 37°C before adding lysis buffer. Equal amounts of cell lysate were analyzed by SDS-PAA gel-electrophoresis Western blotting, using mouse β1-integrin antibodies. Next, blots were stripped and probed for β-tubulin.

**Figure 2:**
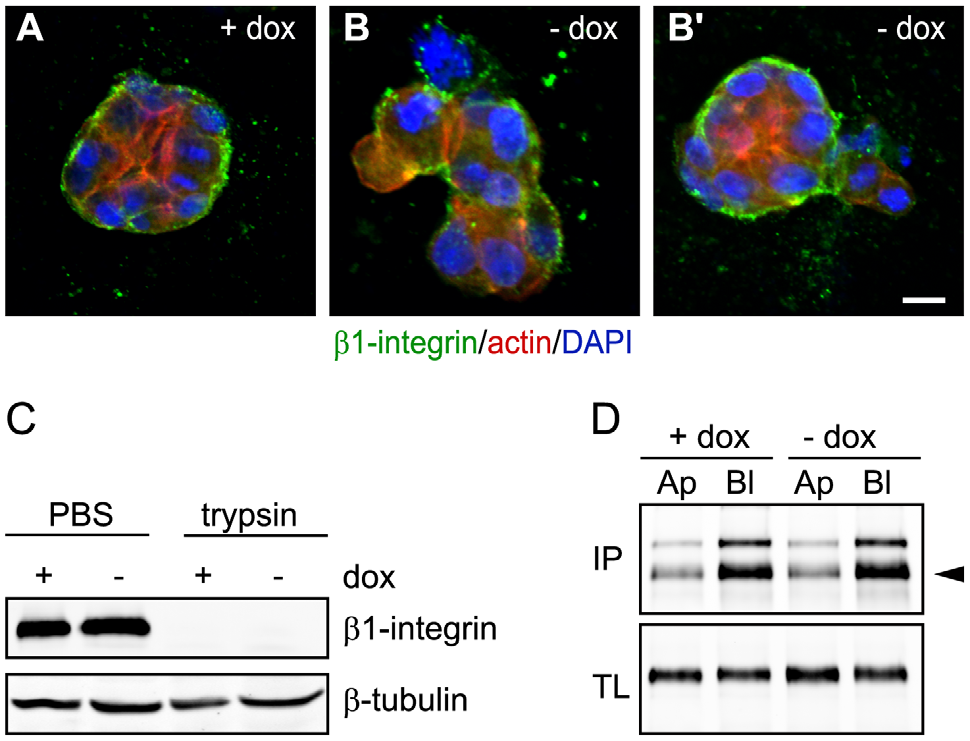
β1 integrin localization, synthesis and transport in Pak1-L107F expressing cells. **A–B’**: Control (A) or CA-Pak1-expressing cells (B,B’) were grown in 3D collagen for 4 days and stained for F-actin (red) and β1-integrins (green). Scale bar is 10 µm. **C**: Control (+dox) and CA-Pak1-expressing cells (-dox) were grown on Transwell filters for 6 days and extracellular proteins were removed by mild trypsinisation. Western blots show untreated, total (PBS) and intracellular (trypsin) levels of β1 integrin as determined by a trypsin protection assay. **D**: Control (+dox) and CA-Pak1-expressing cells (-dox) were grown on Transwell filters for 6 days. Levels of apical (Ap) and basolateral (Bl) β1 integrin were determined by cell surface biotinylation, followed by Western blotting as shown in top panel (IP). Arrowhead shows mature β1 integrin. Levels of β1 integrin in total lysates (TL) are shown in bottom panel.

### Biotinylation Experiments

Surface-specific biotinylation experiments for β1 integrin were done as described [26]. Briefly, cells were grown for 6 days on filters and washed 3x with PBS^++^. Cells were incubated 2×15 minutes with PBS^++^ containing 500 µg/ml EZ-Link Sulfo-NHS-Biotin (Pierce Biotechnology) at the apical or basolateral side of the Transwell. Cells were washed 5 times with 50 mM NH_4_Cl in PBS^++^ to quench unreacted biotin. Next, cells were lysed in IP lysis buffer and β1 integrin was immunoprecipitated with rat monoclonal antibody AIIB2 as described above. Samples were analyzed by SDS-PAA gel-electrophoresis, transferred to PVDF and the blots were probed with IRDye 800CW Streptavidin (LI-Cor, Lincoln, NE). Blots were stripped and reprobed for β1 integrin using mouse anti-β1 integrin antibody.

### Confocal Fluorescence Microscopy

To stain paxillin and pY397-FAK, cells were briefly extracted (60 sec) with CSK buffer (50 mM NaCl, 300 mM sucrose, 3 mM MgCl_2_, 10 mM Pipes, pH 6.8, 0.5% (v/v) Triton X-100) on ice prior to fixation in 4% paraformaldehyde in PBS for 5 minutes at RT, followed by a 10 minute fixation in methanol at –20°C. Samples were blocked and permeabilized with 5% normal donkey serum, 0.1% Triton X-100 in PBS for 60 minutes, and incubations with primary and secondary antibodies were done in the same buffer. Samples were mounted in FluorSave™ (Calbiochem) supplemented with 10 µg/ml DAPI to stain nuclei. For all other 2D stainings, samples were fixed in 4% PFA for 20 minutes at room temperature, followed by the staining procedures described above. Cells in 3D culture were fixed and stained as previously described [23]. Samples were imaged on a Zeiss 510 LSM confocal microscope with an Axiovert 200 M microscope and a C-Apochromat 63x/1.2 W Corr lens. Images showing single confocal slices were cropped and adjusted for brightness with Adobe Photoshop CS4 version 11.0.2 and composite images with scale bars were made with Adobe Illustrator CS4 version 14.0.0.

### Live Cell Imaging

Time-lapse imaging was done on a Zeiss 510 LSM confocal microscope outfitted with a Pecon XL3-LSM temperature and CO_2_ controlled chamber. Cells, grown in the presence (control) or absence (Pak1 mutant expression) of doxycycline were resuspended in a collagen I matrix as described [23] and 150 µl of cells in collagen I was plated in a 4-compartment CELLview glassbottom dish 35,0/10 mm (Greiner Bio-One). Time-lapse differential interference contrast images were captured on the transmitted light detector using the Multi-time macro in the Zeiss 510 LSM software, with 10 minute-intervals. Time-stamped montages of images were prepared with ImageJ.

**Figure 3:**
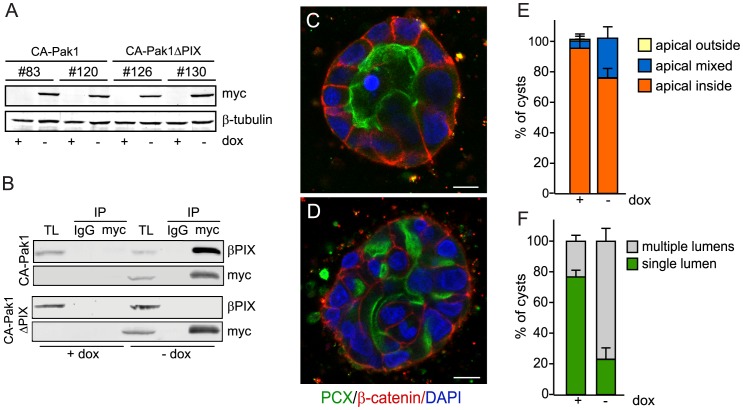
PIX binding is required for CA-Pak1-induced defects in apical orientation, but not lumen formation. **A**: Western blot showing expression levels of different clones of CA-Pak1 and CA-Pak1ΔPIX cells grown with or without dox. Lysates were from 6-day old confluent cultures. **B**: Endogenous βPIX co-immunoprecipitates with CA-Pak1 but not CA-Pak1ΔPIX. Ectopically-expressed active Pak1 mutants (-dox) were immunoprecipitated with anti-myc monoclonal antibodies and associated β-PIX was detected by Western blotting with anti-β-PIX. Immunoprecipitations using anti-mouse IgG were used as negative controls. Total lysate (TL). **C–D**: Control cells (C, +dox) or cells expressing CA-Pak1ΔPIX (D, -dox) were grown in 3D collagen I for 4 days. Cells were stained for PCX (green), β-catenin (red) and nuclei (blue). Scale bar is 10 µm. **E**: Quantification of orientation of apical surfaces in spheroids described in C and D was determined as described in the legends of Figure 1H. Data show means ± SEM. n = 3. **F**: Quantification of spheroids with single (green) or multiple (grey) lumens. Data show means ± SEM. n = 3.

### Statistical Analysis

Statistical significance was determined by a paired *t* test. P values less than 0.05 were considered significant.

## Results

### Constitutive Active Pak1 Deregulates Orientation of the Apical Surface and Inhibits Lumen Formation in 3D Culture

β1 integrin-mediated cell-ECM adhesion promotes the formation and orientation of the apical surface in 3D cultures of MDCK cells via downstream activation of Rac1 [8], [14], [15]. Consequently, constitutive active Rac1 rescues the reversed polarity and lack of lumens in cysts in which β1-integrins are inhibited [8]. To test if this depends on downstream activation of Pak1, we tested whether an active Pak1 mutant could rescue the β1-integrin-inhibitory phenotype as well. For this, we used the Pak1-L107F mutant which is constitutive active because of a mutation in its autoinhibitory domain [27]. We generated MDCK cells that inducibly express this mutant, hereafter called CA-Pak1, under control of the tetracycline-regulated transactivator using the Tet-off system [28]. In this system doxycycline (+dox) inhibited expression, whereas removal of doxycycline induced expression of CA-Pak1 (-dox) (Figure 1A, myc staining). Activation of Pak1 was confirmed by increased phosphorylation of MEK1 on Ser298 (Figure 1A), a site that is specifically phosphorylated by group A Pak kinases [29].

**Figure 4:**
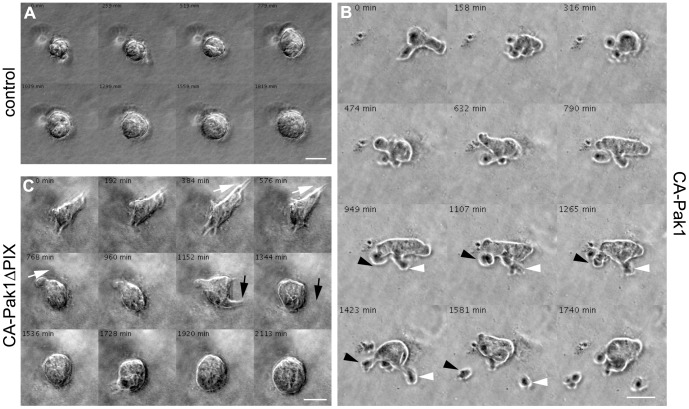
Constitutive active Pak1 promotes cell invasion in 3D collagen culture in a PIX-dependent manner. **A–C**: Control cells (A), or cells expressing CA-Pak1 (B) or CA-Pak1ΔPIX (C) were plated in a 3D collagen I matrix and were imaged for 42–53 h. Images were taken every 11 minute, and are shown as Movies S1, S2, S3. Images show time-lapse intervals of 259 min (control), 158 (CA-Pak1) or 192 min (CA-Pak1ΔPIX) of the spheroids 29–35 h after plating. Arrowheads in CA-Pak1 cells (B) show cells that detach from the main spheroid body and invade into the collagen. Arrows in CA-Pak1ΔPIX (C) cells show transient filapodia-like protrusions. Scale bars are 50 µm.

We analyzed 4 day-old cysts, which, when grown in collagen I, are still immature and in the process of forming apical lumens [30]. Polarization and lumen formation depends on the recruitment of atypical PKC to apical junctions in many epithelia [31], [32]. Consistent with this, PKCζ accumulated at apical junctions that surround the developing lumens in control cysts (Figure 1B,B’). In contrast, PKCζ localized peripheral in CA-Pak1-expressing cysts (Figure 1C,C’), suggesting defects in polarization. Indeed, staining cysts for the basolateral marker β-catenin and the apical marker podocalyxin (PCX)/gp135 revealed that control cysts had a single apical lumen (Figure 1D) but that this organization was disrupted in CA-Pak1-expressing cells, which formed irregularly shaped cell clusters that often had an protruding apical surface oriented towards the matrix (Figure 1E). In addition, many of these clusters had small apical lumens (arrowheads in Figure 1E). Because these cell clusters lacked large lumens and are thus technically not cysts, we will here refer to these clusters as spheroids. While the CA-Pak1 expressing spheroids often appeared larger as compared to controls, quantification of the percentage of DAPI-stained cells with mitotic figures revealed no significant difference between control and CA-Pak1-expressing cells (1.9%±0.7 vs. 3.2% ±0.8, respectively, n = 7, p = 0.21).

**Figure 5:**
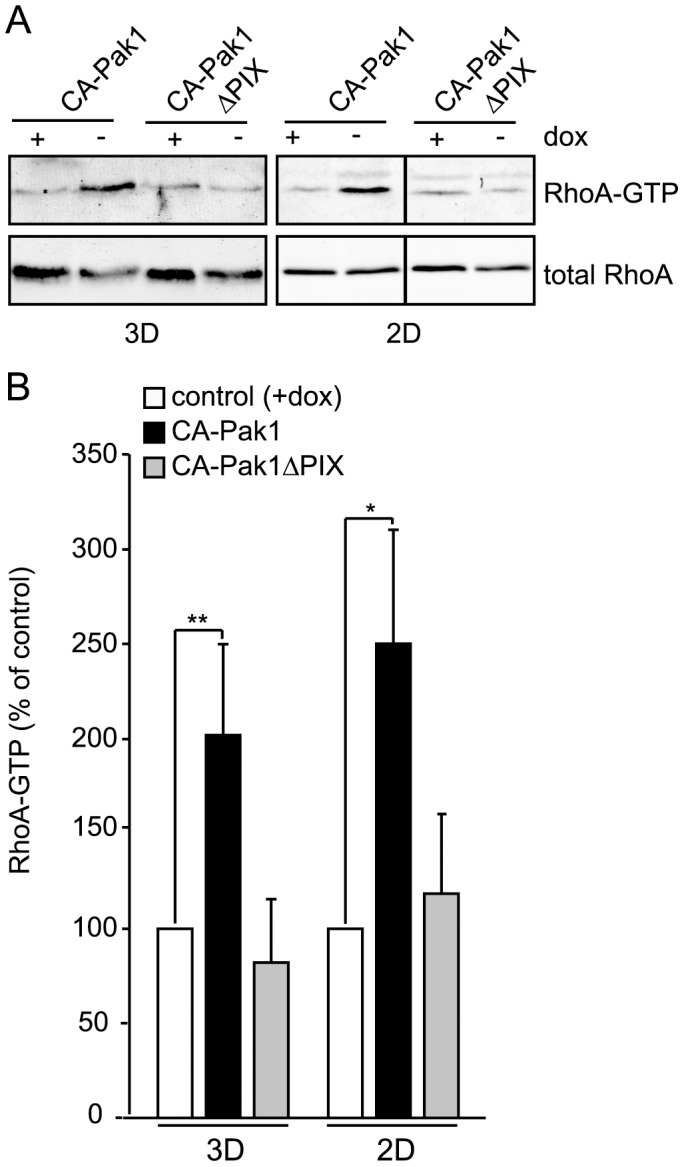
Activation of RhoA in 3D and 2D culture. **A**: Cells were grown in the absence or presence of dox for 24 h prior to plating in a collagen I matrix (3D) or as confluent monolayers (2D), and grown for an additional two days. Western blots show levels of RhoA.GTP and total RhoA. B: Quantification of RhoA.GTP levels of cells expressing CA-Pak1 (black bars) or CA-Pak1ΔPIX (grey bars) in 2D and 3D as percentage of control (+dox, white bars). Data represent mean ± SEM. n = 3 (3D) or n = 4 (2D), **p<0.01, *p<0.02.

**Figure 6:**
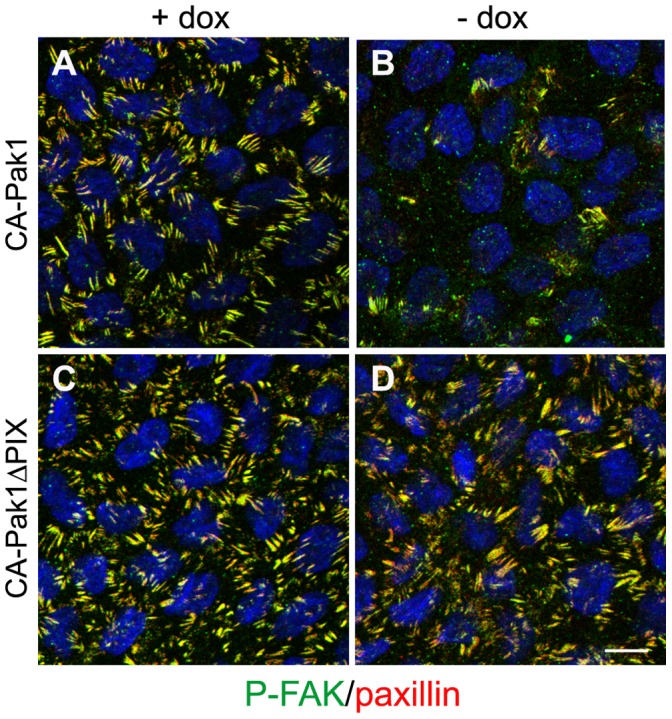
Dissolution of focal adhesions by CA-Pak1. A–D: Confluent control cells (A,C, +dox) and cells expressing CA-Pak1 (B) or CA-Pak1ΔPIX (D) were plated on glass coverslips and stained for P-Y397-FAK (green), paxillin (red) and nuclei (blue). Images show confocal images from the basal surface and nuclei, which locate above this focal plane are therefore only partially visible. Scale bar is 10 µm.

Inhibition of β1 integrins with the function blocking antibody AIIB2 inverts apical polarization and blocks lumen formation in spheroids (Figure 1F, [8]), and activation of Rac1 in this context restores the polarization defect [8]. In contrast, CA-Pak1 did not recapitulate this rescue but rather increased the level of tissue disorganization of AIIB2-treated spheroids (Figure 1G, arrowhead indicates small lumen). Quantification (Figure 1H) revealed that ∼70% of untreated control spheroids had a normal polarization, which we classified as *apical inside* (orange,) meaning that the apical surface is exclusively oriented towards the interior (e.g., as seen in Figure 1D). Of the remaining control spheroids, we classified 29% to have a “*apical mixed”* phenotype (blue), meaning that apical surfaces were oriented towards (small) lumens as well as the matrix-facing exterior (e.g., as seen in Figure 1E,G). Less than 2% of spheroids were classified as *apical outside*, meaning that they lacked lumens and had apical surfaces oriented exclusively towards the exterior (yellow, e.g., as seen in Figure 1F). Either the inhibition of β1 integrin, or the expression of active Pak1 resulted in spheroids that almost exclusively had an *mixed polarity* or *outside apical* phenotype. In combination, the percentage of spheroids with an *outside apical* surface increased (Figure 1H). Together, these data indicate that constitutive activation of Pak1 inhibits the normal orientation of the apical pole. Knockdown of Pak1 with siRNA oligonucleotides did not grossly affected apical polarization in 3D (compare a scrambled control siRNA in Figure 1I to siRNA to Pak1 in Figure 1J). Furthermore, expression of a dominant-negative Pak1 mutant (Pak1-K299R), which likely inhibits the function of all type A Paks by inhibiting their turnover at focal adhesions [33], also did not affect polarization ([23] and data not shown). Instead, as we showed previously, this mutant induces the formation of tubules in older cultures [23].

**Figure 7:**
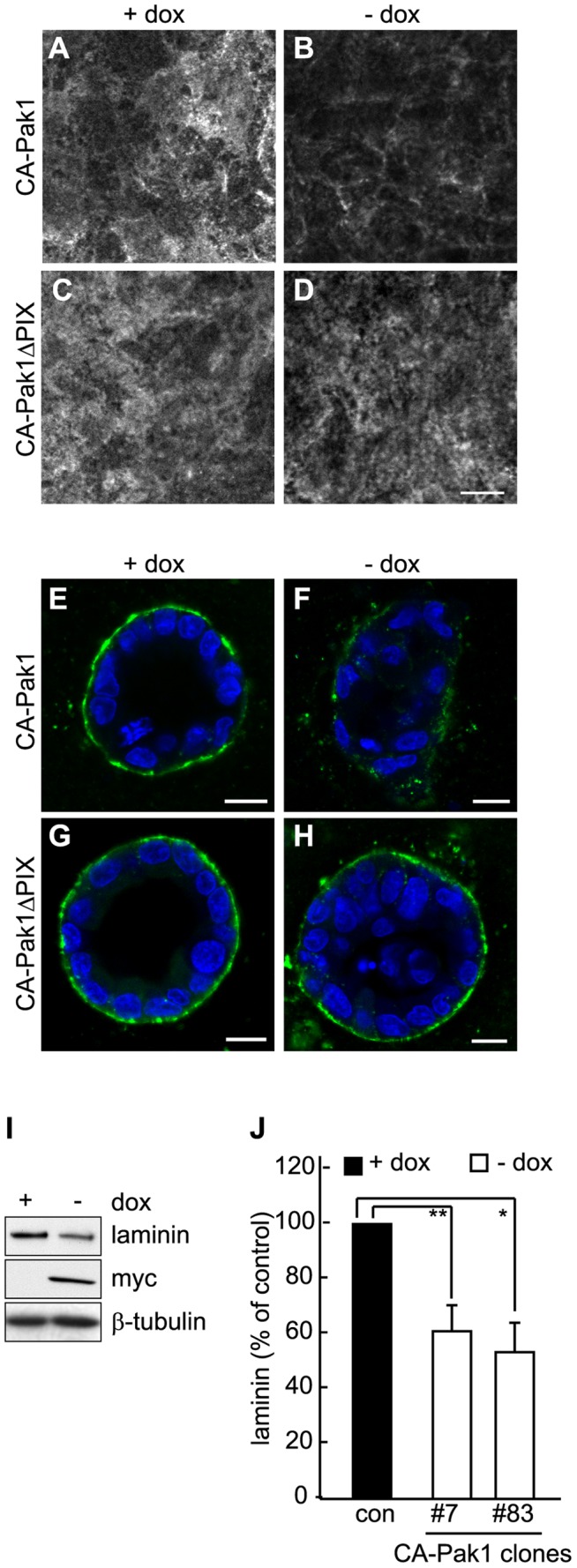
CA-Pak1 inhibits laminin deposition. **A–D**: Controls (+ dox, A,C) and cells expressing CA-Pak1 (B) or CA-Pak1ΔPIX (D) were grown on Transwell filters for 6 days. Cells were fixed and stained for laminin underneath the basal surface. **E–H**: Controls (+ dox, E,G) and cells expressing CA-Pak1 (F) or CA-Pak1ΔPIX (H) were grown in 3D collagen I for 5 days. Cells were fixed and stained for laminin (green). Nuclei, stained with DAPI, are blue. Scale bars in A–H are 10 µm. **I**, Western blot of total laminin levels of 2D lysates from control (+dox) and CA-Pak1 expressing cells (-dox). Myc shows expressing levels of CA-Pak1, β-tubulin is loading control. **J**, Quantification of total laminin in two different Pak-L107F clones. Data represent mean ± SEM. n = 3 for #7 and n = 5 for #83. **p<0.02, *p<0.05.

### CA-Pak1 does not Act by Inhibiting β1 Integrin Transport or Localization

Since the phenotypes of spheroids expressing CA-Pak1 resembled that of spheroids in which β1 integrins were inhibited, we next tested whether CA-Pak1 affected the localization of β1 integrins. Control and CA-Pak1 expressing spheroids exhibited prominent staining of β1 integrins at the basal surface (Figure 2A,B,B′; β1 integrin in green), but the protruding (apical) surfaces in the CA-Pak1 spheroids were mostly devoid of β1 integrins. To investigate if the β1 integrins were correctly trafficked to the plasma membrane, we analyzed the levels of intracellular β1 integrins in filter-grown cells, using mild trypsinization to digest extracellular β1 integrins. This treatment protected intracellular proteins from digestion, as shown by unaltered levels of β-tubulin, but removed all detectable β1 integrins in both control and induced cells. This confirms that most β1 integrins were located at the plasma membrane under both conditions (Figure 2C). MDCK cells were reported to express a small pool of β1 integrins at their apical surface at levels that are too low to be detected by immunofluorescence [11]. To test if CA-Pak1 increased this pool by missorting β1 integrins, we biotinylated proteins at either the apical and basolateral surface, and analyzed apical and basolateral integrins as described in the Methods. Consistent with a previously report [11], we detected an apical pool of β1 integrins, but this pool was not affected by expression of CA-Pak1 (Figure 2D). Finally, Western blot analysis showed that the total protein expression of α3 (data not shown) and β1 integrins (Figure 2C,D) was not altered upon expression of CA-Pak1. Taken together, our data indicate that the CA-Pak1-induced phenotype is not due to a gross defect in transport of β1 integrins to the plasma membrane.

**Figure 8:**
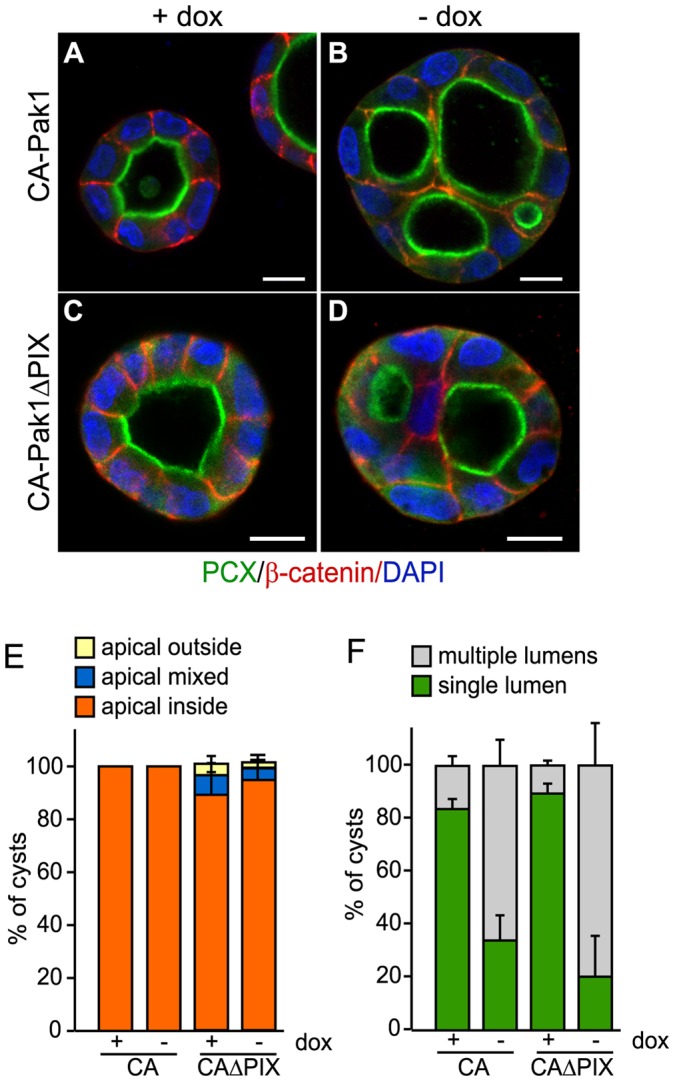
Endogenous matrix or BME rescues the orientation of polarization but not single lumen formation. **A–F**: Cells expressing CA-Pak1 (A,B) or CA-Pak1ΔPIX (C,D) were grown in BME for four days before staining for PCX (green in A–D), β-catenin (red in A–D) and nuclei (blue in A–D). Scale bars are 10 µm. **E–F**: Quantification of apical orientation (F) and lumen formation (G) of cysts shown in A–D. Data show means ± SEM. n = 3.

**Figure 9:**
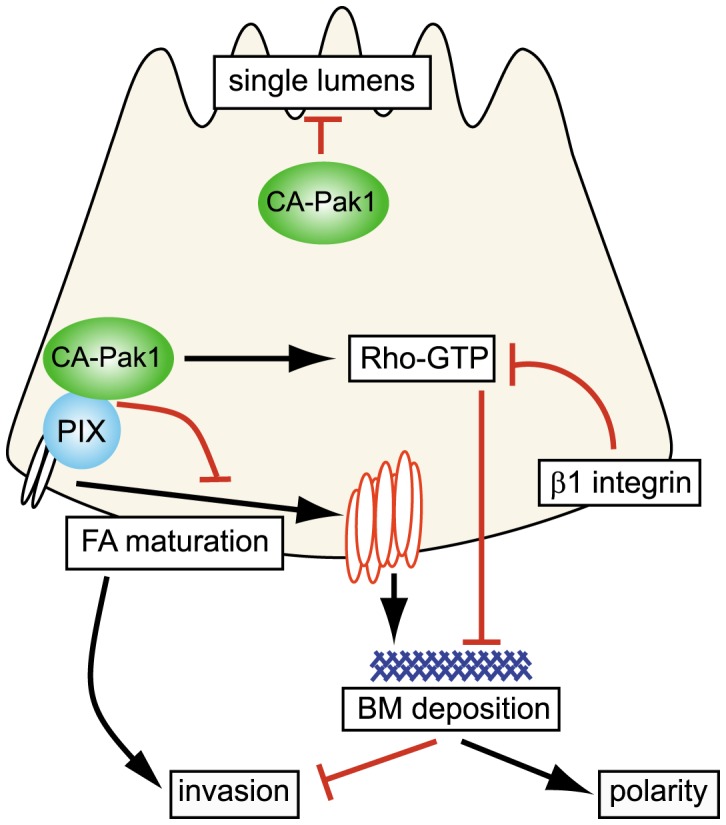
Model for distinct roles of Pak1 in epithelial morphogenesis. *Bottom left to right:* CA-Pak1 is recruited by PIX to immature focal complexes and promotes their turnover, which inhibits cell motility and migration in a collagen I matrix and may inhibit the assembly of a basement membrane. CA-Pak1 also stimulates RhoA activation in a PIX-dependent manner, and this could inhibit basement membrane formation by a mechanism similar to what is seen in cells where β1-integrin function is inhibited [14]. The combined effects of CA-Pak1 on migration and the basement membrane could inhibit apico-basolateral polarization and promote invasion. *Top*: CA-Pak1 inhibits the formation of single lumens by a process that does not depend on its interactions with PIX.

### The Interaction of CA-Pak1 with PIX is Required to Misorient the Apical Surface, but not for the Defects in Lumen Formation

Paks are recruited to cell-ECM adhesion sites by interacting with the Pak-interacting Rac guanine-nucleotide exchange factor (PIX) [34]. To test if the CA-Pak1-induced phenotype depends on its interaction with PIX, we generated cells that inducibly express a myc-tagged CA-Pak1 mutant in which we introduced two additional point mutations that abolish PIX binding (Pak1-L107F,R193G,P194A), hereafter called CA-Pak1ΔPIX [34]. We generated different stable clones with expression levels similar to the CA-Pak1-expressing cells (Figure 3A) and showed that βPIX binding was abolished by immunoprecipitating the myc-tagged CA-Pak1 and CA-Pak1ΔPIX with myc antibodies, followed by Western blotting for βPIX (Figure 3B). When grown in 3D culture, we found that CA-Pak1ΔPIX did not inverse apicobasolateral polarization (Figure 3C–E), but still exhibited a multilumen phenotype (Figure 3D,F).

### Activation of Pak1 is Sufficient for Cell Invasion

The anti-adhesive glycoprotein PCX has functional roles in organizing the apical surface. Its levels are upregulated in many carcinomas [35], which correlates with metastatic potential of renal carcinomas [36]. The CA-Pak1-expressing spheroids were often characterized by cells that stained intensely for PCX, in particular at large rounded areas of the plasma membrane that protruded into the surrounding matrix (see Figure 1E). To analyze these protrusions in greater detail we generated time-lapse movies of 3-day old live cysts over a time course of 2 days, so that at the end of the movie the cysts were 5 days old (Figure 4, Movies S1, S2, S3). Control cysts (+dox) increased in size but retained a circular shape at all times (Figure 4A, Movie S1). Additionally, they demonstrated extensive and persistent rotational movements, as was observed before [37], [38] (Movie S1). In contrast, spheroids expressing CA-Pak1, and in particular the rounded areas protruding into the matrix, were highly dynamic. Often, these areas represented individual cells or small clusters of cells, that eventually detached from the spheroid and invaded the surrounding matrix in an amoeboid fashion (Figure 3B, see arrowheads, Movie S2). We rarely observed rotational movements in CA-Pak1-expressing spheroids (Movie S2 and not shown). The highly dynamic invading phenotype required the interaction of CA-Pak1 with PIX, as expression of CA-Pak1ΔPIX completely abolished detachment of cells. Instead, transient filopodia-like structures were seen (Figure 3C, see arrows, Movie S3). The CA-Pak1ΔPIX-expressing spheroids sometimes moved as a cohort up to distances of 100 µm in 24 h (not shown), and rotational movements were also observed. Together, our data suggest that expression of CA-Pak1 is sufficient to drive cell invasion and this depends on its interaction with PIX.

### CA-Pak1 Increases RhoA Activation but Inhibits Focal Adhesions

The similarities in the CA-Pak1 phenotype and the phenotype induced by β1 integrin inhibition, may suggest that CA-Pak1 acts on downstream signaling intermediates of this pathway. Hyperactivation of RhoA is crucial for inverting apical polarization upon inhibition of β1 integrins [14]. In CA-Pak1-expressing spheroids the levels of active RhoA (RhoA.GTP) were increased about 2-fold, whereas CA-Pak1ΔPIX (Figure 5A,C) did not affect RhoA activation. RhoA activation generally promotes the maturation of focal adhesions and the formation of stressfibers [39]. As these are difficult to image in 3D culture, we analyzed these structures in cells grown on coverslips. Also at these conditions, CA-Pak1, but not CA-Pak1ΔPIX increased the activation of RhoA (Figure 5A,B). Surprisingly however, immunostaining for paxillin and active focal adhesion kinase (phospho-Y397-FAK) revealed that CA-Pak1 (Figure 6, compare A to B) caused a dramatic decrease in the number of focal adhesions, and an accompanying loss of stressfibers (data not shown). This phenotype was not seen in cells expressing CA-Pak1ΔPIX (Figure 6, compare C to D).

### Pak1 is Involved in Organizing Basement Membrane

Cell-matrix interactions are sufficient to orient the apical pole of epithelial cells in 2D culture [1]. The deposition and organization of heterotrimeric laminin molecules into a basement membrane-like structure is required for proper orientation of the apical domain and lumen formation in embryoid bodies [7] and in 3D cultures of MDCK and mammary epithelial cells [8], [15], [40]. Cell-matrix adhesion receptors regulate laminin organization [41], and a loss of focal adhesions may thus inhibit this process. To detect extracellular deposited laminin we stained non-permeabilized CA-Pak1-expressing cells grown on Transwell filters or in 3D culture for laminin. For this, we used polyclonal antibodies against laminin-111, which stain the β1 and γ1 chains of laminin-511 in MDCK cells [8], [15]. Co-staining cells for the intracellular protein p120catenin did not yield any signal, thus showing that only extracellular proteins were detected with our fixation and staining procedure. CA-Pak1, but not CA-Pak1ΔPIX, caused a dramatic decrease in deposited laminin in both 2D (Figure 7A–D) and 3D (Figure 7E–H) culture. This was at least partially due to decreased synthesis or stabilization, as Western blot analysis of two different CA-Pak1-expressing clones showed a modest but consistent reduction in laminin levels in 6-day old cultures (Figure 7I,J).

### Basement Membrane Rescues Polarity Orientation, but not Lumen Formation

To test if the decreased laminin deposition caused the CA-Pak1-induced phenotype, we plated cells in reconstituted basement membrane (BME), which contains high levels of laminin. In BME, control cells polarized faster as compared to collagen (compare 4 day-old cysts in collagen in Figure 1D,H to cysts in BME in Figure 8A,E). BME completely rescued the polarity phenotype of CA-Pak1-expressing cells (Figure 8B,E). Interestingly however, BME did not rescue the defects in single lumen formation in either these cells (Figure 8B,F) or in the CA-Pak1ΔPIX cells (Figure 8E,F), although the lumens were generally larger in BME as compared to collagen I (compare Figure 8E to 3D). Together, these data suggest that defects in basement membrane organization upon CA-Pak1-expression induces misorientation of apical polarization, but that the defects in single lumen formation are mediated by another mechanism.

## Discussion

Inhibition of β1 integrins in MDCK cysts grown in 3D culture causes misorientation of the apical surface and inhibits lumen formation via Rac1 and its downstream role in laminin assembly [8]. Our previous data support a model in which β1 integrin-mediated activation of Rac1 inhibits RhoA-dependent ROCK1 activation and actin-myosin contractility. Decreased actin-myosin contractility, in turn, is required for a proper assembly of a basement membrane, which drives normal morphogenesis. Thus, inhibition of β1 integrins leads to increased RhoA-dependent actin-mysosin contractility, inhibition of laminin assembly into a basement membrane and subsequent defects in polarization and lumen formation [8], [14]. Consistent with this model, expression of constitutively active Rac1 in the context of β1 integrin inhibition rescues the assembly of laminin and the aberrant phenotype.

Herein, we test if this rescue by Rac1 relies on its effector molecule Pak1. We used the constitutive active mutant Pak1-L107F instead of the previously commonly used active loop mutant Pak1-T423E, as it was recently shown that this mutant is not constitutively active [42], and because of our previous findings that this mutant acts as a dominant-negative in MDCK cells [21], [33]. We find that expression of a constitutive active mutant of the Rac1 effector Pak1 reverts polarization and inhibits single lumen formation, and induces a phenotype that resembles β1 integrins inhibition. Using antibody staining for activated Pak1, we did not find that inhibition of β1 integrin leads to an activation of Pak1 (data not shown). Our data therefore indicate that Pak1 is not a direct signaling intermediate in the β1 integrin-Rac polarity orientation pathway. Rather, we show that at least some Pak1-mediated signaling routes converge with the integrin-Rac-Rho-pMLC pathway at the level of Rho activation. Furthermore, constitutive active Pak1 appears to act through at least partially distinct pathways to control polarity orientation and lumen formation, respectively (Figure 9). Thus, misorientation of the apical surface and ensuing cell invasion is associated with activation of RhoA and misassembly of laminin. This process depends on the interaction of active Pak with PIX, which recruits Pak1 to cell-matrix adhesion sites [34], [43]. In contrast, the role of constitutive active Pak1 on lumen formation is at least partially independent of the Pak-PIX interaction, and not associated with RhoA activation.

The large decrease in laminin assembly into a basement membrane in CA-Pak1 cells is accompanied by a dramatic decrease in focal adhesions. The loss of focal adhesions did not inhibit apical polarization of cells on Transwell filters, although these cells exhibited multilayering and formed intercellular apical lumens between multilayered cells (data not shown). Under these conditions, the CA-Pak1 cells were more proliferative (data not shown). This was in contrast to cells in 3D culture, where proliferation was not significantly affected, thus ruling out that increased proliferation is the driving force behind the loss of tissue architecture in these cells. As integrins have been widely implicated in the deposition of laminin [41] we propose that the decrease of laminin deposition and formation of focal adhesions in CA-Pak1 expression are functionally linked. Inhibition of Paks increases the size and/or number of focal adhesions [21], [44], [45], [46]. This suggests that Pak1 activity is required for turnover of these structures (reviewed in [47]), but the mechanisms are not entirely clear. Pak1 can regulates focal adhesion directly, via phosphorylation of the focal adhesion proteins paxillin [48] and GIT1 [49], or indirectly, through Pak-mediated regulation of actin-myosin contractility or microtubule dynamics [50]. Regardless of the mechanism however, our data support a model in which Pak1-dependent dissolution of focal adhesions inhibits the assembly of a basement membrane, which in turn is required to properly polarize cells and maintain tissue integrity (Figure 9).

Pak organizes the fibronectin matrix in Xenopus embryos via a mechanism that relies on the regulation of cellular tension and α5β1 integrins [51] and it is possible that cellular tension also plays a role in the assembly of laminin into a basement membrane in our model. Indeed, we previously showed that increased RhoA activation and actin-myosin contractility inhibits basement membrane deposition in MDCK cysts downstream of β1 integrin [14]. As RhoA activation is generally associated with an increase in stressfibers and focal adhesions, it was surprisingly to find that active Pak1 dramatically decreased these structures (stressfibers not shown), even though it increased RhoA activation. Nevertheless, the cellular phenotype we observe here resembles the loss of tissue architecture in epithelial tumors [52] and amoeboid cell migration [53] that has been associated with increased RhoA activity. It is possible that RhoA is activated at a different subcellular site than the cell-ECM contacts. Although our attempts to identify the subcellullar localization of RhoA activation have been unsuccessful thus far, an appealing possibility would be that this occurs near the apical surface, at sites where PCX accumulates. PCX can form a complex with NHERF1 and ezrin, which causes an ezrin-dependent activation of RhoA and reorganization of the apical actin cytoskeleton in MDCK cells [54]. βPIX can also be recruited into this complex and may be required for the metastatic behavior of PCX-overexpressing renal carcinomas [36]. It is thus possible that Pak1 is recruited via βPIX and regulates RhoA activation via this complex. Alternatively, Pak1 may activate RhoA via RhoGEF GEF-H1, as has been described previously [55].

In contrast to the polarization defects, the multilumen phenotype in CA-Pak1 cells did not depend on the interaction of the mutant with βPIX or the presence of a basement membrane, and also does not seem to be associated with RhoA activation. Therefore, we propose that CA-Pak1 controls lumen formation by a mechanism that is at least partially distinct from apical polarization orientation. These findings are consistent with our previous work in which we showed that Rac1 could rescue apical polarization defects caused by β1 integrin inhibition, but did not rescue the formation of single lumens [8]. A precedent for Pak1 in lumen formation was shown in the Drosophila salivary glands, where CA-Pak1 interferes with normal lumen formation by inducing the formation of multiple intercellular lumens. This lumen defect is caused by increased endocytosis of E-cadherin [19]. In our system, adherens junctions, as judged by the localization of β-catenin (Figure 1E and 4) and E-cadherin (not shown), are not directly affected in cells expressing CA-Pak1 (not shown) or dominant-negative Pak1 [21], [23], [33]. We therefore consider it unlikely that the lumen defect results from a loss of E-cadherin function. Another underlying cause of multilumen phenotypes are defects the orientation of the mitotic spindle, which can be controlled by PKCζ [56], [57]. Pak1 can constitutively associate with PKCζ [58], and Pak2 controls the orientation of the spindle, albeit in a βPIX-dependent manner [59]. Nevertheless, as we show that Pak1 mislocalizes PKCζ, it is feasible that Pak1 controls formation of single lumens by regulating the mitotic spindle.

In conclusion, we show here that Pak1 regulates tissue architecture via different, matrix-dependent and independent mechanisms and that defects in orientation of the apical surface can be partially uncoupled from defects in lumen formation. Future efforts will be dedicated in deciphering if and how Pak1 controls invasion by local Rho activation and whether defects in spindle orientation cause defects in lumen formation.

## Supporting Information

Movie S1 **Control cells in 3D collagen I.** Movie corresponding to montage in Figure 3A. Time-lapse images of control cells in a 3D collagen I matrix. Images were taken every 11 minutes.
(AVI)

Movie S2 **CA-Pak1 cells in 3D collagen I.** Movie corresponding to montage in Figure 3B. Time-lapse images of CA-Pak1-expressing cells in a 3D collagen I matrix. Images were taken every 11 minutes.
(AVI)

Movie S3 **CA-Pak1ΔPIX cells in 3D collagen I.** Movie corresponding to montage in Figure 3C. Time-lapse images of Pak1-L107F R193G,P194A-expressing cells in a 3D collagen I matrix. Images were taken every 11 minutes.
(AVI)
